# Tilted Nano-Grating Based Ultra-Compact Broadband Polarizing Beam Splitter for Silicon Photonics

**DOI:** 10.3390/nano11102645

**Published:** 2021-10-08

**Authors:** Haipeng Liu, Jijun Feng, Jinman Ge, Shanqing Zhuang, Shuo Yuan, Yishu Chen, Xiaojun Li, Qinggui Tan, Qinghua Yu, Heping Zeng

**Affiliations:** 1 Shanghai Key Laboratory of Modern Optical System, Engineering Research Center of Optical Instrument and System, Ministry of Education, School of Optical-Electrical and Computer Engineering, University of Shanghai for Science and Technology, Shanghai 200093, China; Liuhaipeng232@163.com (H.L.); 193720544@st.usst.edu.cn (S.Z.); ysh183882148@163.com (S.Y.); chenyishu199651@163.com (Y.C.); 2 National Key Laboratory of Science and Technology on Space Microwave, China Academy of Space Technology, Xi’an 710100, China; gjm129@163.com (J.G.); lxjzhxl@163.com (X.L.); uestctqg@163.com (Q.T.); 3 Key Laboratory of Intelligent Infrared Perception, Shanghai Institute of Technical Physics, Chinese Academy of Sciences, Shanghai 200083, China; yuqinghua2000@126.com; 4 Chongqing Key Laboratory of Precision Optics, Chongqing Institute of East China Normal University, Chongqing 401120, China; hpzeng@phy.ecnu.edu.cn; 5 State Key Laboratory of Precision Spectroscopy, East China Normal University, Shanghai 200241, China

**Keywords:** nano-grating, polarizing beam splitter, silicon photonics, integrated photonics

## Abstract

An ultra-compact broadband silicon polarizing beam splitter is proposed based on a tilted nano-grating structure. A light cross coupling can be realized for transverse-magnetic mode, while the transverse-electric light can almost completely output from the through port. The length of the coupling region is only 6.8 μm, while an extinction ratio of 23.76 dB can be realized at a wavelength of 1550 nm. As a proof of concept, the device was fabricated by a commercial silicon photonic foundry. It can realize a 19.84 dB extinction ratio and an 80 nm working bandwidth with an extinction ratio of larger than 10 dB. The presented device also shows a good fabrication tolerance to the structure deviations, which is favorable for its practical applications in silicon photonics.

## 1. Introduction

The photonic integrated circuit is considered as one of the key technologies for large-capacity, low-latency, and high-speed optical communication networks [1,2,3]. The silicon-on-insulator (SOI) platform has aroused considerable interest due to its high refractive index contrast enabling compact footprint and the compatibility with complementary-metal-oxide-semiconductor (CMOS) technology, facilitating its low-cost mass production. However, its high index contrast inevitably causes the polarization-dependent loss as well as the corresponding waveguide dispersion. Thus, polarization control and manipulation are crucial for the device design. As one of the key polarization-handling devices, a polarizing beam splitter (PBS) has always attracted much attention, which can separate the orthogonally polarized transverse-electric (TE) and transverse-magnetic (TM) light into different pathways [4]. Different structures have been demonstrated for on-chip polarization splitting, such as multimode interferometers (MMIs) [5,6] directional couplers [7,8] or Mach–Zehnder interferometer [9], etc. An MMI coupler can be used for PBS but with a long length, due to its weak polarization dependence intrinsically [10]. Mach–Zehnder interferometer-based PBSs have also been reported, while additional metal heaters or complex fabrication processes are needed [11]. Conventional directional couplers are widely used due to the low insertion loss but suffering from a narrow bandwidth and tight fabrication tolerance [12]. Moreover, an ultra-compact PBS is always favorable for practical applications as the chip integration density can be increased. Plasmonic [13], slot [14], and photonic crystal waveguide [15] assisted directional couplers have been proposed for the ultra-compact PBS. However, they suffer from a relatively large loss and limited working bandwidth. Subwavelength gratings (SWGs) have been widely applied recently, which can behave as homogenous media and effectively suppress the diffraction effects [16,17,18,19]. Effective refractive index of the grating waveguide can be controlled by adjusting the period or duty cycle. The grating-based PBS can achieve a 40 nm bandwidth [20] or an extinction ratio of 15 dB [21]. However, the sizes of the above-mentioned structures are in the scale of tens micrometers. To further decrease the device size and improve the bandwidth performance, a tilted nano-grating based PBS is proposed here. The coupling region is only 6.8 μm, which is the smallest grating type directional coupler-based PBS to the best of our knowledge. 

In the following, a tilted nano-grating structure-based PBS is designed and fabricated. TE light can output from the through port, while the TM light can meet a phase matching condition and output from the cross port. The grating structure such as the tilted angle, width, and duty cycle has been properly optimized, with considering the extinction ratio as well as the insertion loss. A commercially available CMOS compatible manufacturing facility is used for the device fabrication, and a wide working bandwidth can be realized. 

## 2. Structure and Design

**Figure 1:**
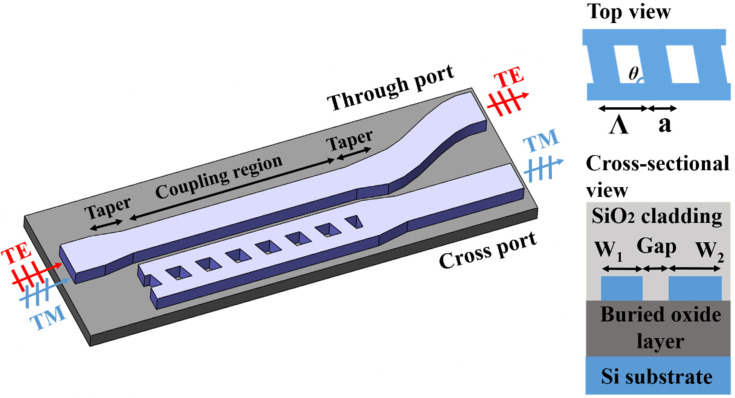
Schematic illustration of the proposed tilted gratings based polarizing beam splitter.

Schematic illustration of the proposed device is shown in Figure 1, which consists of tilted gratings and strip waveguides in the coupling region as shown in the right insets for the top and cross-sectional view. Tapered waveguides are placed at the start and end of the coupling region, as slightly wide input and output waveguides can reduce the bending radius with a low propagation loss. The incident waveguide width is chosen to be 0.5 μm with consideration of the single-mode condition. An S-bend waveguide is used to separate the through and cross-ports. A 220 nm-thick SOI wafer is adopted with a 3 μm-thick silicon dioxide buffering layer and a 2 μm-thick cladding layer. 

The widths of the strip and tilted gratings waveguides are *W*_1_ and *W*_2_, respectively. The grating period is *Λ* with ridge width of *a* and duty cycle of *a/Λ*. The refractive indices are 1.444 and 3.476 for SiO_2_ and Si, respectively. To suppress the diffraction effects, the grating period should be shorter than *λ/*2*n_eff_*, which corresponds to the Bragg condition with *λ* for working wavelength and *n_eff_* for effective mode index. The grating period and duty cycle are chosen to be 400 nm and 0.5 respectively for easy of fabrication. The effective mode index of the SWG waveguide is engineered by changing the SWG widths to realize an effective mode index matching with the strip waveguide for TM mode.

Equivalent medium theory is an efficient way to analyze the SWG waveguide [22]. The SWG waveguide with a short grating period behaves like an equivalent waveguide but with a decreased refractive index and the light propagation can be described as in a homogeneous index-guided structure [23]. The equivalent refractive index can be roughly estimated according to Rytov’s formula for *TE* or *TM* light, as in [24]:

(1) $$n_{TE}^{2}\approx \frac{a}{\Lambda }\cdot n_{Si}^{2}+\frac{\Lambda -a}{\Lambda }\cdot n_{SiO_{2}}^{2},$$

or

(2) $$\frac{1}{n_{TM}^{2}}\approx \frac{a}{\Lambda }\cdot \frac{1}{n_{Si}^{2}}+\frac{\Lambda -a}{\Lambda }\cdot \frac{1}{n_{SiO_{2}}^{2}}$$

**Figure 2:**
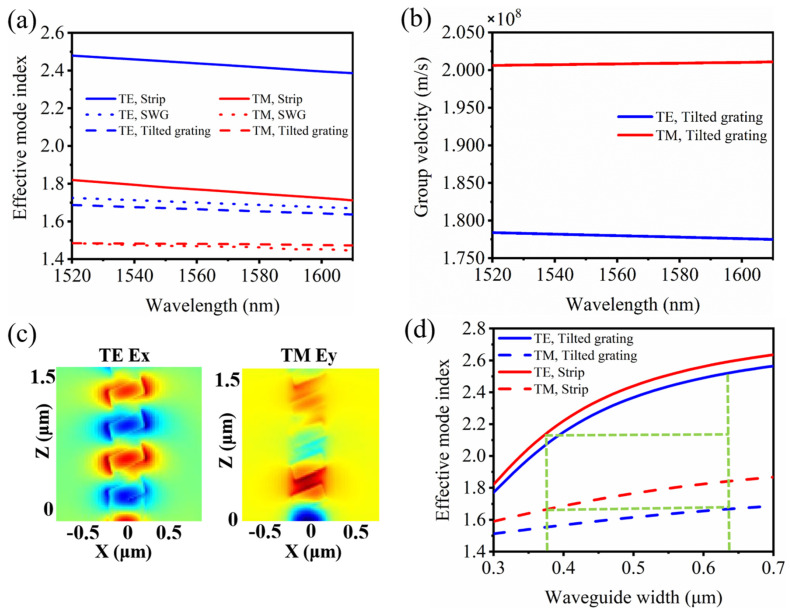
(**a**) Effective mode indices for TE and TM modes of the strip, SWG, and tilted grating waveguides. (**b**) Group velocities of TE and TM modes for tilted grating waveguide. (**c**) Corresponding electric fields for the TE (Ex) and TM (Ey) modes propagating along the tilted gratings. (**d**) Effective mode indices of TE and TM modes for different strip and tilted grating waveguide widths.

Then the effective mode indices of *TE* and *TM* modes in the strip, SWG, and tilted gratings (with a 20°-tilted angle) waveguide with a 500 nm width are simulated based on the finite-element method as in Figure 2a. It can be seen that the change of effective mode index for TM light is only about 0.015 for the tilted grating waveguide, while that is about 0.133 for the strip waveguide or 0.05 for the SWG case in the wavelength range of 1520–1610 nm. It can thus be expected that the tilted grating structure can realize a wide working bandwidth. To further understand the potential broadband characteristics, the group velocity of the tilted grating structure is analyzed as in Figure 2b. It can be seen that in the whole C+L wavelength band, the group velocity only varies 0.3% for TM mode, which can help it to realize a wideband light cross coupling. The corresponding electric fields for the TE and TM modes propagating through the tilted gratings are shown in Figure 2c. 

The waveguide effective mode indices are then calculated at a 1550 nm wavelength for the coupling strip or tilted grating waveguides with varying widths as in Figure 2d. The strip waveguide width *W*_1_ is chosen to be 0.38 μm to realize a phase matching with the tilted grating with width *W*_2_ of 0.64 μm. Too narrow strip waveguide will cause a large propagation loss and need a longer tapered structure for the bending waveguide. On the other hand, too wide strip waveguide will cause a longer coupling length and require a much wider tilted grating and smaller duty-cycle, which will increase the fabrication difficulty. Either the strip or tilted grating waveguide has an effective mode index of about 1.66 for TM light (dashed horizontal line in Figure 2d). While for the TE mode, a large phase mismatching due to the effective mode index difference could prevent power transfer between the strip and tilted grating waveguides, with the corresponding indices of 2.15 and 2.52, respectively.

**Figure 3:**
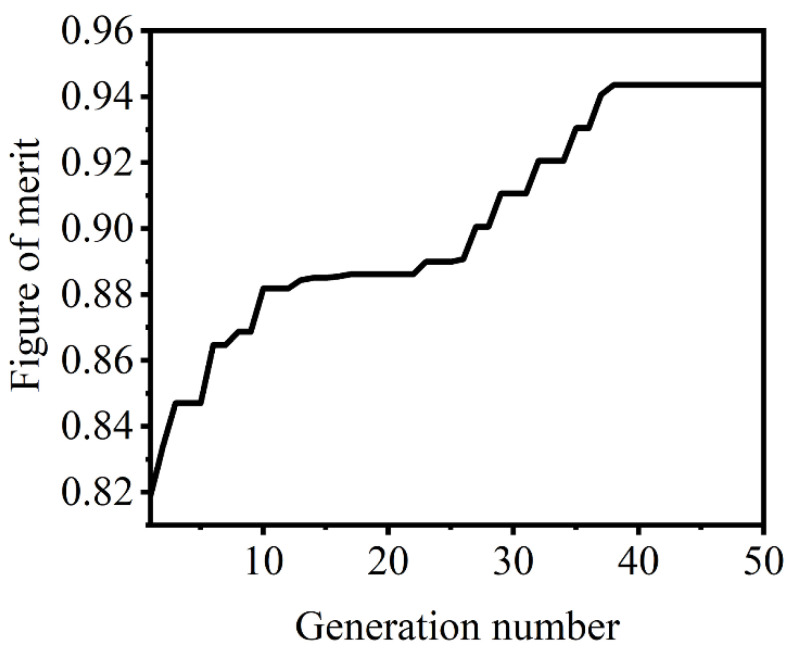
FOM trend for the PSO simulation.

The device performance is further optimized using 3D finite-difference time-domain (FDTD) simulation. With consideration of the compatibility with commercially available 180 nm CMOS compatible facility, the gap between the strip and tilted grating waveguides is chosen to be 220 nm. As the tilted grating waveguide behaving as an equivalent homogeneous waveguide, the PBS can work as a conventional directional coupler. The PBS length is calculated to be 5.8 μm, which can realize a TM cross coupling between the strip and tilted grating waveguides according to the supermode theory [25]. To achieve accurate phase matching for the TM mode, particle swarm optimization (PSO) is applied for automatically searching of the optimum waveguide parameters. The PSO algorithm returns the maximum or the minimum number of a figure of merit (FOM) by searching the structure parameters [26]. The width of the strip waveguide varies from 0.36 to 0.41 μm, while that of the gratings waveguide varies from 0.61 to 0.66 μm. The variation of the side mesa width of the grating waveguide is in the range of 0.19–0.24 μm. Considering the computing efficiency and simulation accuracy, the mesh size is set as 2 nm with a simulation domain from the input strip waveguide to the output bending region. A perfect matching layer is applied with the PSO swam size of 20 and generation number of 50. The average cross-port coupling efficiency for the TM light in the wavelength range of 1520–1610 nm is applied as FOM, which can reach a stable value of about 94.4% after 40-generation optimization as shown in Figure 3. The optimized widths of the strip and tilted grating waveguides are 0.379 and 0.62 μm with those for the side mesa of tilted grating are 0.23 and 0.2 μm, respectively.

**Figure 4:**
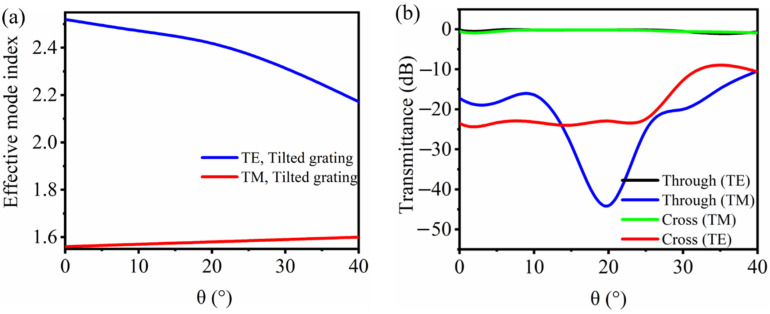
(**a**) Effective mode index for TE and TM modes of the tilted grating waveguide at *λ*_0_ = 1550 nm. (**b**) Simulated transmission spectra for both polarizations under different tilted angles.

Though the grating tilted angle does not change the duty cycle, the effective mode index of the grating waveguide and corresponding light field propagation would be slightly affected as shown in Figure 4a. By using 3D FDTD method with varying grating ridge tilted angle as in Figure 1, the transmission spectra for both output ports under different polarizations are simulated as in Figure 4b. A 20°-tilted ridge can result in a maximum extinction ratio (defined as the transmittance difference between two polarizations at the output port) of about 23.76 dB at a 1550 nm wavelength, while the device has almost no insertion loss. 

**Figure 5:**
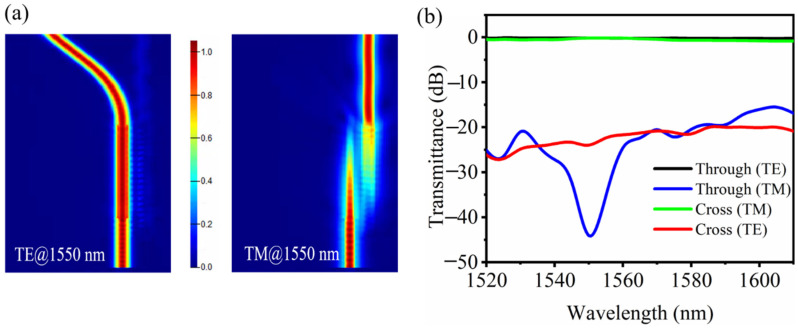
(**a**) Simulated light propagation and (**b**) transmission spectra of the PBS device for both polarizations.

After determining the optimum waveguide structure parameters, a fine optimization to the device coupling length is implemented. The light propagation properties for TE and TM mode with an optimum coupling length of 6.6 μm are shown in Figure 5a. It can be seen that TM light can couple completely to the cross port, while TE light comes out from the through port with almost no light coupling. The simulated transmission spectra of the PBS device for both polarizations are shown in Figure 5b. At a 1550 nm wavelength, the transmittance for TE or TM light at the through port is about −0.20 or −44.07 dB, while that at the cross port is −23.96 or −0.19 dB. A 23.76 dB extinction ratio can be realized here with an extinction ratio of more than 10 dB in the whole C+L wavelength band.

**Figure 6:**
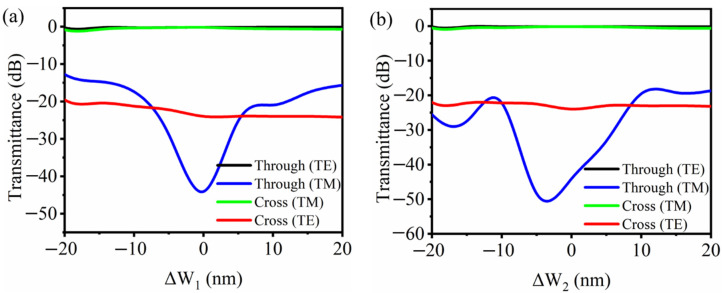
Simulated transmission spectra for both polarizations with the change of waveguide widths (**a**) Δ*W*_1_ and (**b**) Δ*W*_2_.

Fabrication tolerance is also analyzed with consideration of the variation of waveguide width Δ*W*_1_ or Δ*W*_2_. Figure 6a,b show the transmission spectra with the strip and tilted grating waveguide widths changing in the range of −20~20 nm at a 1550 nm wavelength. The device reveals a large tolerance to the fabrication errors, with a lower than 0.56 dB insertion loss as well as a larger than 17.5 dB extinction ratio within a ±10 nm waveguide width variation.

## 3. Fabrication and Characterization

**Figure 7:**
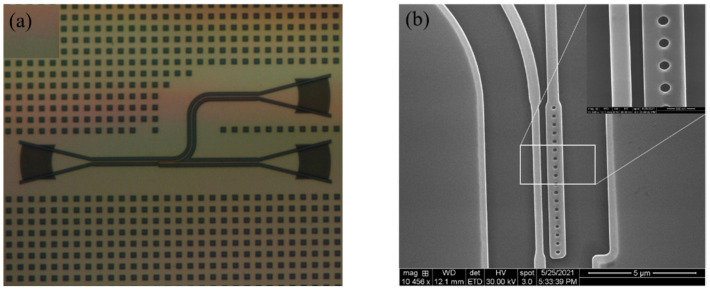
(**a**) Microscope image of the chip under test and (**b**) SEM image of the fabricated device.

As a proof-of-concept, the PBS devices with different coupling lengths were fabricated by using a commercially available 180 nm CMOS compatible fabrication facility. An optimum polarizing beam splitter is realized at a coupling length of 6.8 μm, which deviates slightly from the designed parameters due to the fabrication errors. Figure 7a shows the microscope image of the fabricated chip. The scanning electron microscope (SEM) image of the fabricated device is shown in Figure 7b. The SiO_2_ cladding layer was slightly etched by buffered hydrofluoric acid for clear visualization. It is obvious that the etched shape of the designed tilted grating changes from a parallelogram to a circular hole. The reason may be due to the lithography and etching accuracy, which will slightly affect the device’s performance. 

**Figure 8:**
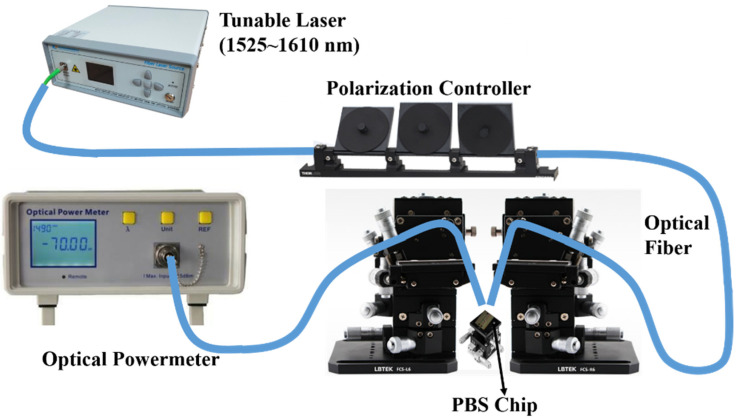
Experimental setup for the device characterization.

**Figure 9:**
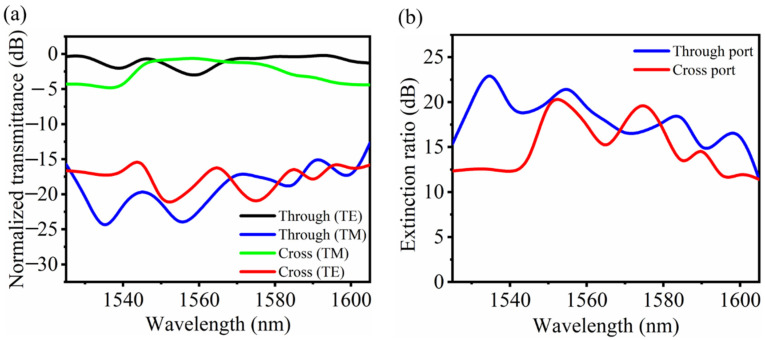
(**a**) Measured normalized transmission spectra of the PBS device and (**b**) corresponding extinction ratio for both polarizations.

For device characterization, a schematic of the experimental setup is illustrated in Figure 8. A tunable laser was used, and the wavelength of the input light was tuned from 1525 to 1605 nm with a step of 2 nm. An optical powermeter was applied as the receiver for the measurement of the transmittance at the output port. Figure 9a shows the measured transmission spectra at the through and cross ports of the fabricated PBS. Here, the measured transmission spectra were normalized with respect to those of the strip waveguide on the same chip, in order to exclude the influence of the grating couplers. The corresponding extinction ratio is shown in Figure 9b, which can reach 19.84 dB at a 1550 nm wavelength. The extinction ratio is more than 10 dB in the whole wavelength range, as expected by the theoretical simulations.

**Figure 10:**
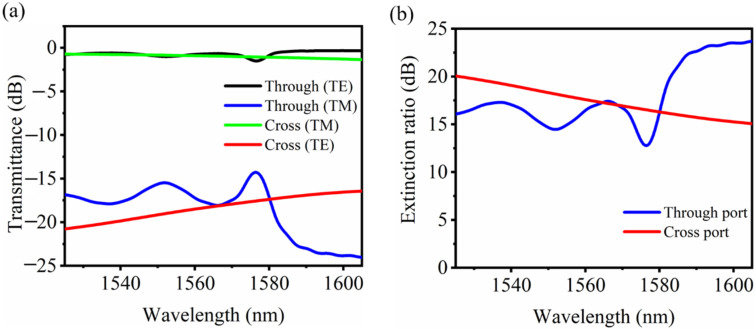
(**a**) Simulated transmission spectra and (**b**) corresponding extinction ratio for both polarizations with the device parameters obtained by the SEM image of the fabricated chip.

The extinction ratio of PBS would be deteriorated with the increase of working wavelength. It is worth noting that the wavelength corresponding to the highest extinction ratio is around 1555 nm, which is 19.92 dB and red-shifted compared to the designed 1550 nm wavelength. This may be caused by the fabrication errors such as the grating ridge shape and size. Actually, we further simulated the device performance according to the chip parameters obtained by the measured SEM image (Figure 7b), with a round hole instead of the tilted grating structure. As shown in Figure 10b, the wavelength corresponding to the highest extinction ratio shifts to 1564 nm, whose extinction ratio is only 17 dB but also greater than 10 dB in the wavelength range of 1525–1605 nm. Though the simulation coincides well with the measured results, the change of grating shape could cause the performance deterioration. It should be noted that the transmission spectra for both output ports are stable for TE light at different working wavelength, as little light coupling happens. While for TM light, the transmission spectrum for the through port jitters with the wavelength, coinciding well with the experimental results.

We further summarize a comparison of the presented device and some other reported grating structure-based SOI PBSs as in Table 1. It can be seen that the current PBS can realize a high extinction ratio and a broad working bandwidth with an ultra-compact footprint. Meanwhile, the current device was prepared using a commercially available fabrication facility, which would favor its low-cost manufacturing. With a higher precision lithography processing, a tilted structure can be expected, which could help to improve the device performance.

## 4. Conclusions

To summarize, a wideband and compact silicon polarizing beam splitter was demonstrated based on the tilted nano-grating waveguide structure. TM light can couple completely to the cross port, while TE light can output mainly from the through port. The presented device shows a wide working bandwidth with favorable tolerance to manufacturing deviations. By commercially available fabrication facility, the device was manufactured and could achieve an 80 nm bandwidth with extinction ratio of larger than 10 dB, though the tilted grating structure was only roughly formed. The fabricated device has a coupling length of only 6.8 μm, with an extinction ratio of 19.84 dB at a 1550 nm wavelength. The presented broadband polarizing beam splitter is expected to have a wide application prospect in silicon photonics and related communication networks.

## Figures and Tables

**Table 1:** Comparison of different types of PBSs reported recently.

Structures	Extinction Ratio	Insertion Loss	BW_10 dB_ *	Length	Year
Grating assisted couplers [17]	21 dB	0.48 dB	40 nm	19 μm	2015
Asymmetric coupler with SWG [15]	15 dB	2.6 dB	60 nm	12 μm	2017
MMI coupler with SWG [16]	20 dB	2.5 dB	84 nm	100 µm	2018
Side-wall tilted SWG [18]	15 dB	1 dB	72 nm	14 μm	2020
This work	23.76 dB	0.2 dB	80 nm	6.8 μm	

* BW_10 dB_ is the bandwidth with extinction ratio of greater than 10 dB.
